# The genetics of Ug99 stem rust resistance in spring wheat variety ‘Linkert‘

**DOI:** 10.3389/fpls.2024.1343148

**Published:** 2024-03-07

**Authors:** Erena A. Edae, Zennah Kosgey, Prabin Bajgain, Kimani C. Ndung'u, Ashenafi Gemechu, Sridhar Bhavani, James A. Anderson, Matthew N. Rouse

**Affiliations:** ^1^Department of Plant Pathology, University of Minnesota, Saint Paul, MN, United States; ^2^Kenya Agricultural and Livestock Research Organization (KALRO), Food Crops Research Centre, Njoro, Kenya; ^3^Department of Agronomy and Plant Genetics, University of Minnesota, Saint Paul, MN, United States; ^4^Ethiopian Institute of Agriculture, Debre Zeit Agricultural Research Center, Bishoftu, Ethiopia; ^5^International Maize and Wheat Improvement Center (CIMMYT), Texcoco, Mexico; ^6^Cereal Disease Laboratory, United States Department of Agriculture-Agricultural Research Service, Saint Paul, MN, United States

**Keywords:** QTL, stem rust, wheat, disease resistance, 90K iSelect

## Abstract

Wheat stem rust caused by *Puccinia graminis* f. sp. *tritici (Pgt)* threatens wheat production worldwide. The objective of this study was to characterize wheat stem rust resistance in ‘Linkert’, a variety with adult plant resistance effective to emerging wheat stem rust pathogen strain Ug99. Two doubled haploid (DH) populations and one recombinant inbred line (RIL) population were developed with ‘Linkert’ as a stem rust resistant parent. Hard red spring wheat variety ‘Forefront’ and genetic stock ‘LMPG’ were used as stem rust susceptible parents of the DH populations. Breeding line ‘MN07098-6’ was used as a susceptible parent of the RIL population. Both DH and RIL populations with their parents were evaluated both at the seedling stage and in the field against *Pgt* races. Genotyping data of the DH populations were generated using the wheat iSelect 90k SNP assay. The RIL population was genotyped by genotyping-by-sequencing. We found QTL consistently associated with wheat stem rust resistance on chromosome 2BS for the Linkert/Forefront DH population and the Linkert/MN07098-6 RIL population both in Ethiopia and Kenya. Additional reliable QTL were detected on chromosomes 5BL (125.91 cM) and 4AL (*Sr7a*) for the Linkert/LMPG population in Ethiopia and Kenya. Different QTL identified in the populations reflect the importance of examining the genetics of resistance in populations derived from adapted germplasm (Forefront and MN07098-6) in addition to a genetic stock (LMPG). The associated markers in this study could be used to track and select for the identified QTL in wheat breeding programs.

## Introduction

Stem rust of wheat (*Triticum aestivum* L.), caused by *Puccinia graminis* f. sp. *tritici* (*Pgt*) Erikss. & Henning, is a destructive disease of wheat worldwide (Singh et al., 2011; Savary et al., 2019). The emergence of a race of *Pgt* known as Ug99 that defeats the widely deployed stem rust resistance gene, *Sr31*, poses a threat to wheat production (Singh et al., 2015). The use of genetic resistance is a preferred strategy for mitigating losses from crop diseases including stem rust of wheat.

Breeding for disease resistance is one of the primary objectives of wheat improvement programs in the United States and worldwide. Since the early 20^th^ century, there have been international collaborations on genetic resistance breeding for controlling stem rust disease in wheat (*Triticum* spp.) (Carleton, 1905; Waldron, 1935; Stakman, 1955; Evans, 1980). Consequently, to date 64 stem rust resistance (Sr) genes have been characterized and mapped to chromosome locations in wheat (McIntosh et al., 2020). Out of these, 16 wheat Sr genes have been cloned by either map-based cloning (*Sr13, Sr21, Sr33, Sr35, Sr50, Sr55, Sr57, Sr60*, and *Sr62*) or target sequence capture approaches (*Sr22, Sr26, Sr27, Sr43, Sr45*, *Sr46*, and *Sr61*) (Arora et al., 2019; Periyannan et al., 2013; Zhang et al., 2017; Chen et al., 2018; Saintenac et al., 2013; Mago et al., 2015; Moore et al., 2015; Krattinger et al., 2009; Steuernagel et al., 2016; Chen et al., 2020; Zhang et al., 2021; Yu et al., 2022). Since the discovery of Ug99, there has been an intensive search for resistance genes both in domesticated wheat and its wild relatives, and 36 numerically designated stem rust resistance genes that are effective to the Ug99 race group have been characterized (Rouse et al., 2014a; Singh et al., 2015): *Sr2, Sr12, Sr13, Sr14, Sr15, Sr21*, *Sr22, Sr25, Sr26, Sr27, Sr28, Sr29, Sr32, Sr33, Sr35, Sr37, Sr39, Sr40, Sr42, Sr43, Sr44, Sr45, Sr46, Sr47, Sr48, Sr50, Sr51, Sr52, Sr53, Sr55, Sr56, Sr57*, *Sr59*, *Sr61, Sr62*, and *Sr63*. However, only few are available in conventional North American spring wheat germplasm including *Sr2, Sr12, Sr13, Sr25, Sr55*, and *Sr57* (Rouse et al., 2014b; Bajgain et al., 2015a; Edae et al., 2018). In addition, many QTL have also been reported using bi-parental populations (Hiebert et al., 2010; Haile et al., 2012; Njau et al., 2013; Zurn et al., 2014; Kumsa et al., 2015; Bajgain et al., 2015b), and association mapping (Yu et al., 2011; Letta et al., 2013; Zhang et al., 2014; Bajgain et al., 2015a; Edae et al., 2018). Despite the availability of numerous genes and QTL, stem rust still remains a threat to wheat production worldwide due to continuous emergence of new *Pgt* races in different parts of the world and the lack of resistance deployed in conventional wheat varieties effective to the emerging races (Olivera et al., 2015; Newcomb et al., 2016; Olivera et al., 2019, 2022). This implies the importance of persistently carrying out resistance breeding in wheat through the identification and characterization of new resistance genes from both domestic germplasm and its wild relatives.

The development of new sequencing technologies has facilitated the discovery of a large number of SNP markers for many crop species such as hexaploid wheat (Chapman et al., 2015), barley (Mascher et al., 2013), rice (Spindel et al., 2013) and maize (Romay et al., 2013). Genomic/genomic resources such as wheat reference sequences The International Wheat Genome Sequencing Consortium (IWGSC), 2018), wheat 90K iselect SNP chips (Wang et al., 2014) and genotype-by-sequencing (GBS), a reduced representation genotyping platform (Poland et al., 2012), have been used effectively to discover genes/QTL for several traits including disease resistance in wheat.

Genetic resistance to rust pathogens can be generally grouped into two categories: resistance that is effective at all plant growth stages and resistance that is effective at the adult plant stage. All-stage resistance genes often confer major-effects and account for thirty of the thirty-six Ug99-effective genes. Adult plant resistance genes often confer relatively minor effects and include *Sr2*, *Lr67/Sr55/Yr46/Pm46*, *Sr56, Lr34/Sr57Yr18/Pm38*, *Lr46/Sr58/Yr29/Pm39*, and *Sr63* (Mago et al., 2002; Singh et al., 2015). These six designated adult plant resistance genes have not been reported to exhibit race-specificity supporting an expectation (Singh et al., 2011, 2014) that effective adult plant resistance genes are non-race-specific. Though few examples of race-specific adult plant resistance genes have been reported for stripe rust (*Yr11*, *12*, *13*, and *14*; Milus et al., 2015) and leaf rust (*Lr12*; Singh and Bowden, 2010), the generally non-race-specific characteristic of adult plant resistance genes justifies an emphasis on adult plant resistance for achieving durable resistance to stem rust in wheat.

Though an abundance of genetic sources of resistance exist to emerging stem rust races, few conventional wheat varieties possess effective resistance. Singh et al., 2015 reported that only 16% of U.S. hard red spring wheat varieties and breeding lines showed resistance to the Ug99 race group. This susceptibility is significant as the U.S. hard red spring wheat growing region is historically vulnerable to major epidemics of wheat stem rust when virulent races are present, resulting in over 50% statewide yield losses in Minnesota and North Dakota during epidemic years. One exception to this widespread vulnerability is hard red spring wheat variety ‘Linkert’, released in 2013 by the University of Minnesota (Anderson et al., 2018). Linkert exhibited consistent adult plant resistance to Ug99 in Kenya (Anderson et al., 2018). Linkert became a successful variety that was grown on 833,900 acres in Minnesota and North Dakota in 2018 and was the most widely grown wheat variety in Minnesota from 2016 to 2020. The objective of this study was to characterize the genetics of stem rust resistance in ‘Linkert’ with particular attention to the adult plant resistance exhibited to Ug99.

## Materials and methods

### Mapping populations

Two doubled haploid (DH) mapping populations were developed by crossing Linkert with both hard red spring wheat variety ‘Forefront’ (Glover et al., 2013) and genetic stock LMPG-6 (‘Little Club’//3 3 ‘Prelude’/8 3 Marquis’/3/’Gabo’), a highly rust susceptible, day length insensitive line that was developed for the purpose of producing near-isogenic lines for *Sr* genes (Knott, 2000). ‘Linkert’ is a hard red spring wheat developed and released by the Minnesota Agricultural Experiment Station (MAES) in 2013. Linkert is one of the highest quality hard red winter wheats with yield comparable to other high protein varieties and better disease resistance. Doubled haploid progeny were produced at a service facility at Washington State University through a modified maize pollination method (Niu et al., 2014). A recombinant inbred line (RIL) population was derived through single seed descent to the F_6_ generation after crossing Linkert and University of Minnesota breeding line ‘MN07098-6’. A total of 107, 190, and 172 lines were derived from the Linkert/Forefront (LK/FF), Linkert/LMPG-6 (LK/LM), and Linkert/MN07098-6 (09X149) populations, respectively.

### Disease evaluations

The lines in the DH populations with their parents were evaluated in the field for three years (2016-2018) in Kenya for response to the Ug99 race group (*Pgt* races including TTKSK, TTKTT), and in Ethiopia for response to *Pgt* races TKTTF, TTKSK, TRTTF and JRCQC (Kosgey et al., 2021). The RIL population (F_6:8_ generation) was evaluated in Kenya and Ethiopia for two years (2016 and 2017). The DH populations were also evaluated at Rosemont, MN for three years (2015-2017) with four domestic *Pgt* races evaluated in single race nurseries inoculated with races QTHJC, QFCSC, TPMKC and RCRSC, according to previously described methods (Edae et al., 2018). Mixtures of spores of *Pgt* races for field evaluations were inoculated both in Ethiopia and Kenya (Kosgey et al., 2021). Stem rust severity was visually scored in the experimental plots based on the modified Cobb scale of 0-100, where 0 = immunity (no uredinia or any other sign of infection) and 100% = completely susceptible (Peterson et al., 1948). Infection response was rated as resistant (R), small uredinia surrounded by necrosis; moderately resistant (MR), medium-sized uredinia surrounded by necrosis or chlorosis; moderately susceptible (MS), medium-sized uredinia without necrosis; susceptible (S), large uredinia without necrosis; or MRMS, an infection response that included both the MR and MS categories (Roelfs et al., 1992). Coefficient of infection (COI) values were generated by multiplying the stem rust severity value for each line by a constant value for each infection response: 0 = 0, R = 0.2, RMR = 0.3, MR = 0.4, M = 0.6, MS = 0.8, S = 1.0 (Knott, 1989). Average coefficient of infection for the two replicates were determined and used for analyses. Raw mean coefficients of infection values across the two randomized replicates in each environment were used for QTL analyses.

At the seedling stage, the DH populations and parents were evaluated with *Pgt* races QTHJC (isolate 0069MN399), QFCSC (isolate 95MN1080), TPMKC (isolate 74MN1409), RCRSC (isolate 00MN99C), TRTTF (06YEM34-1), and TKTTF (isolate 13ETH18-1) according to previously described methods (Hundie et al., 2019). All four parents were susceptible as seedlings to race TTKSK (04KEN156/04; Ug99 race group).

### DNA extraction and genotyping

Tissues were harvested from approximately two weeks old seedlings of the populations and parents and grown in a greenhouse before placing into 96-well plates. The harvested leaves were dried in a lyophilizer and ground for 2 min using a Genogrinder (SPEX Sample Prep). DNA extraction was carried out following an SDS method: 300 µl extraction buffer (200 mM Tris-HCL pH 8.0, 250 mM NaCl, 25 mM EDTA, 0.5% SDS, ddH20) was added to each well followed by gently shaking the 96-well plates before adding 300 µl of chloroform:isoamyl alcohol (24:1) to each well. The suspension was mixed for 3 minutes on a plate shaker followed by centrifuging for 20 min at 2500 rpm, and the supernatant (200-300 µl) was transferred to separate tubes.

After decanting the supernatant, 300 µl of 70% ethanol was added to each tube, the tubes were centrifuged for 20 min at 2,500 rpm, the ethanol was poured out, and the DNA pellets were air-dried. When the pellets were completely dry, 100 µl of 1xTE was added to each tube to resuspend the pellet. DNA was quantified using a NanoDrop 1000 spectrophotometer (Thermo Fisher, Waltham, MA, United States).

Genotyping data of the DH populations were generated using the iSelect 90k SNP assay developed for wheat (Wang et al., 2014). SNPs with minor allele frequency (MAF) less than 25% were removed, all monomorphic markers and markers with missing data points for one or both parental genotypes were also removed. No heterozygote genotypes were retained in the data set. Missing data points were represented by “-”. SNPs with > 10% missing data points were removed, and none of the lines were removed as missing data for all individuals for a given SNP was < 1%. SNPs with segregation distortion p-values of less than 0.5 were removed before creating linkage maps for both DH populations. For the LK/FF population, one SNP was removed due to high missing values in the segregation distortion filtered data set. However, for the LK/LM population, all SNPs had missing values < 6.4% and none of the SNPs were removed. SNPs with lod error (Lincoln and Lander, 1992) values greater than four were removed for the final linkage map construction. After applying the filtering criteria described above, a total of 1,091 and 1,552 high quality SNPs were used for QTL mapping for LK/FF and LK/LM populations, respectively. These SNP markers were used to make linkage groups using JoinMap 4.0 (Upadhyaya, 2006). The Kosambi mapping function was used to estimate the map distance.

Genomic DNA from the Linkert/MN07098-6 population was extracted using the BioSprint 96 DNA Plant Kit (QIAGEN, Valencia, CA) and genotyped using the genotype-by-sequencing approach (Poland et al., 2012). Reads were filtered for phred quality score (Q) of ≥ 30 and de-multiplexed using ‘sabre’ (https://github.com/najoshi/sabre, accessed 05-12-2022). Filtered reads were aligned to the *Triticum aestivum* v1.0 reference sequence (The International Wheat Genome Sequencing Consortium (IWGSC), 2018) using default parameters in the Burrows-Wheeler Aligner 0.7.5 (Li, 2013). Discovery of genome-wide markers (SNPs) was done using default parameters in SAMtools 1.6 and BCFtools 1.6 (Li et al., 2009; Li, 2011). SNPs with heterozygote level and missing data points greater than 10% and 25%, respectively, were removed. A filtered data set comprised of 1,674 SNPs was used for QTL mapping.

Quantitative trait loci (QTL) analysis was conducted in R package RQTL (Broman et al., 2003) with composite interval mapping (CIM) method using both seedling infection type data and field response data from the populations. Before QTL mapping, categorical field infection responses and seedling infection types were converted to a linear 0-9 scale (Zhang et al., 2014; Gao et al., 2016).

## Results

### Phenotypic evaluation

**Figure 1:**
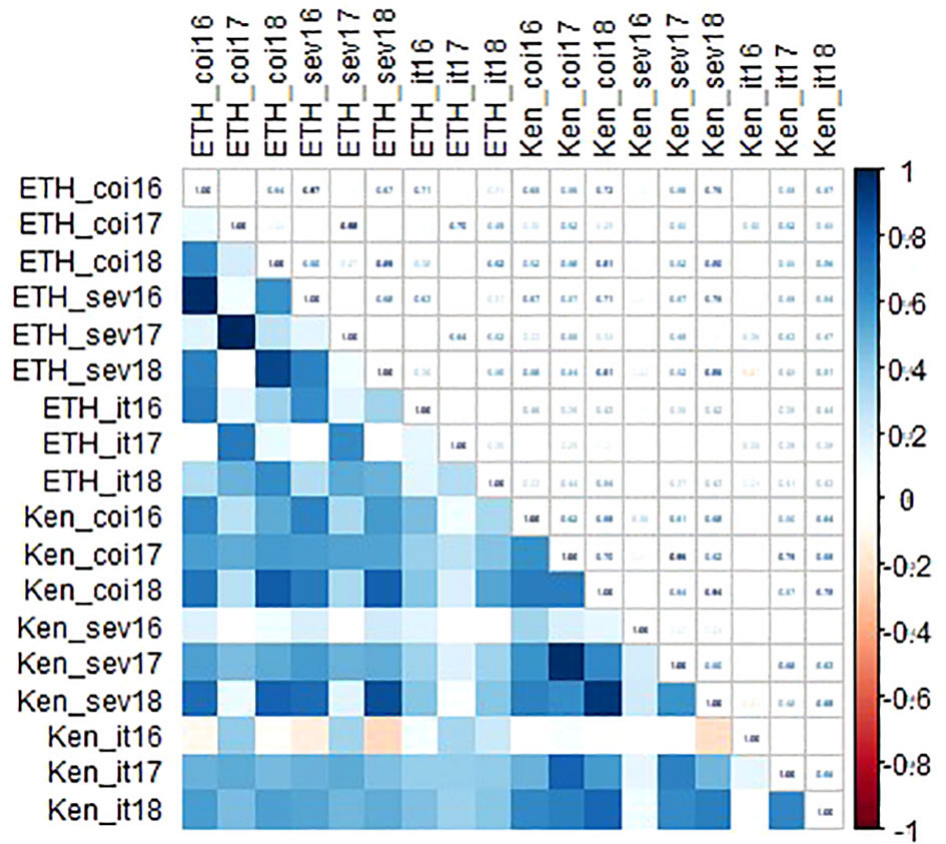
Correlation coefficients among environments for both disease severity and infection response data recorded in Kenya and Ethiopia from 2016-2018.

Linkert was susceptible at the seedling stage to virulent *Pgt* races such as TTKSK, TTKTT, and TTRTF, but was resistant to the Ethiopian TKTTF isolate. Forefront was also susceptible to both TTKSK and TTKTT (**Table 1**). Disease severity values ranged from 1- 47%, 4 - 44% and 4 - 25%, 65 - 70% for Linkert, Forefront, LMPG-6 and MN07098-6, respectively, for response to *Pgt* races in East Africa (**Table 1**). Although LMPG-6 had lower average severity percentage than Linkert and Forefront, the average coefficient of infection was similar for all the three parents (16.26- 17.24). For North American *Pgt* races, Linkert was resistant to all *Pgt* races (QFCSC, RCRSC, QTHJC and TPMKC) tested at the seedling stage whereas LMPG-6 was susceptible to all races. However, Forefront was resistant to QFCSC and RCRSC but susceptible to QTHJC and TPMKC races at the seedling stage (**Table 2**). There was strong phenotypic correlation between Ethiopia and Kenya field data based on severity and infection response except for the data from Kenya in 2016 and Ethiopia in 2017. Both severity and infection response data recorded in 2016 in Kenya were poorly correlated with other years in Kenya and Ethiopia (**Figure 1**; **Supplementary Table 1**).

**Table 1:** Disease severity (%) recorded for Linkert, Forefront, LMPG-6 and MN07098-6 to different stem rust races at different locations (Rosemount, Kenya and Ethiopia).

Race	Variety *			MN07098-6	Locations
	Linkert	Forefront	LMPG-6		
Mixture of *Pgt* races + Ug99 variants	21.6 (1.0 - 47.0)	22.5 (4.0 – 44.0)	13.75 (4.0 – 25.0)	65.0 - 70.0	Kenya and Ethiopia
TPMKC	11.8 (5.0 – 20.0)	13.8 (5.0 - 20.0)	57.5 (55.0-60.0)	–	Rosemount
QTHJC	9.4 (7.5 – 10.0)	21.9 (10 - 32.5)	55.63 (50.0-60.0)	–	Rosemount
QFCSC	10.0 (10.0 - 12.5)	13.1 (5.0 - 22.5)	62.5 (57.5 – 65.0)	–	Rosemount
RCRSC	11.3 (10.0 - 12.5)	18.8 (15 - 22.5)	22.5	–	Rosemount

*Number in parentheses are mean of severity records across replications.

**Table 2:** Seedling infection types recorded for Linkert, Forefront, LMPG-6 and MN07098-6 to different stem rust race in controlled greenhouse.

Race	Variety *			MN07098-6	Locations
	Linkert	Forefront	LMPG-6		
TTKSK	3+	3+	3+, 33+	3+	Greenhouse
TKTTF	0;1	0;1	3, 33+	13-, 0;	Greenhouse
TRTT	33+	2	3+	3+	Greenhouse
TPMKC	22+, 23-	3+, 32+	33+	–	Greenhouse
QTHJC	1;, 1	33+, 33-C	3 + 3, 33+	–	Greenhouse
QFCSC	0;	0;	33+	–	Greenhouse
RCRSC	0;1	3+	0;1	–	Greenhouse

*Number in parentheses are mean of severity records across replications.

Seedling infection type (IT) values in linear scale less than six were considered as resistant whereas IT values greater than six were considered as susceptible. All individuals of the Linkert/Forefront population were resistant to *Pgt* races QFCSC and RCRSC. For all races tested in this study the percentage of resistant lines was greater than 50% except for response to race TPMKC which resulted in resistance in only 35.7% of the Linkert/Forefront and 29.4% of the Linkert/LMPG individuals (**Table 3**).

**Table 3:** Number of resistant and susceptible lines based on seedling reaction following inoculation with six *Pgt*.

	Linkert/LMPG-6		Linkert/Forefront	
	Resistant	Susceptible	Resistant	Susceptible
*Pgt* QTHJC	70	91	57	42
*Pgt* QFCSC	149	15	99	0
*Pgt* TPMKC	47	113	41	63
*Pgt* RCRSC	99	55	103	0
*Pgt* TKTTF	85	43	–	–
*Pgt* TRTTF	–	–	39	46

### Genetic linkage maps

**Figure 2:**
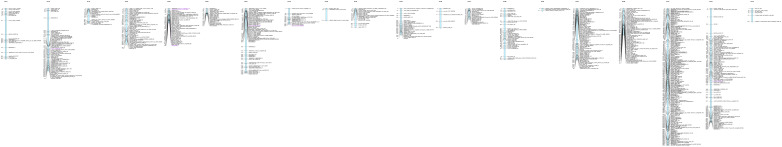
Genetic linkage map based on Linkert/Forefront (LK/FF) population.

**Figure 3:**
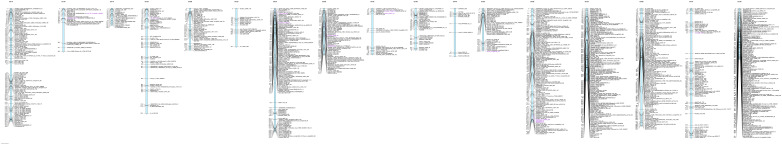
Genetic linkage map based on Linkert/LMPG-6 (LK/LP) population.

For Linkert/Forefront population, a total of 1,088 SNPs from 90k SNP chip mapped to 25 linkage groups, and these 25 linkage groups corresponded to 19 wheat chromosomes. Apart from two wheat chromosomes, 6D and 7D, all wheat chromosomes were represented in the map of Linkert/Forefront population (**Figure 2**). For both populations, chromosome 7B comprised the highest number of SNPs whereas chromosomes 3D (5 SNPs) and 4D (11 SNPs) had the lowest number of SNPs for LK/FF and LK/LM populations, respectively (**Supplementary Tables 2A, B**). For the Linkert/LMPG-6 (LK/LM) population a total of 1,552 SNPs from 90k SNP chip mapped to 25 linkage groups. However, the 25 linkage groups were assigned to 17 wheat chromosomes. Five chromosomes (3D, 5D, 6D and 7D) all were from D genome were missing from the linkage (**Figure 3**; **Supplemental Table 2B**). Since SNP identification was carried out using wheat genome reference sequence for the validation population, Linkert/MN07098-6, linkage map creation was not attempted because we were able to generate this population specific physical map for the 1,674 GBS SNPs (**Figure 3**). Unlike 90K SNP chip, all 21 wheat chromosomes were recovered for the GBS platform and chromosome 6B had the highest number of SNPs (2540) whereas only three SNPs passed the filtering criteria for chromosome 4D (**Supplementary Table 2C**). Relatively GBS SNPs were only evenly distributed on chromosome 6B but for the remaining chromosomes SNPs that met the filtering criteria (see *Materials and Methods* section) were mainly on the terminal ends of chromosomes. However, unlike GBS, large gap size on linkage maps were not observed for the 90K SNP chip most of the cases.

### QTL analyses

In this study, after considering QTL within five centimorgans as redundant QTL, a total of 55 significant QTL distributed on 18 chromosomes were detected for all populations evaluated both under field and seedling stages for resistance to stem rust (**Tables 4**–**7**). QTL were detected on all chromosomes except 5D and 7D. Although the phenotypic variance explained was less than 20% in most cases, the QTL detected on chromosomes 1BL and 4DS in the LK/LM population evaluated in the field for race QFCSC and the QTL on chromosome 4AL for race TKTTF at the seedling stage explained substantial amounts of phenotypic variation (38.4-69.0%). In this study, all-stage QTL were detected only on chromosomes 1B (52.0 cM) and 4AL for race RCRSC detected in the Linkert/Forefront and Linkert/LMPG-6 populations, respectively (**Tables 4**, **5**). The remaining detected QTL were expressed only either at seedling or adult plant stages.

**Table 4:** Adult-stage stem rust QTL detected for Linkert/Forefront (LK/FF) and Linkert/LMPG-6 (LK/LM) populations in Ethiopia and Kenya using coefficient of infection.

Year	QTL name	QTL position (cM)	Chr	Marker @ QTL	Left flanking	Right flanking	LOD	% Variance explained by QTL	Additive effect (a) *	population	Comments
DZ 2017	*QSr.cdl.2BS*	1.86	2B	wsnp_Ku_c48_103915	Tdurum_contig29563_109	GENE-0818_347	8.11	9.67	2.44 (LK)	LK/FF	Kosgey et al. (2021)
DZ 2018	*QSr.cdl.2BS*	1.86	2B	wsnp_Ku_c48_103915	Tdurum_contig29563_109	wsGENE-0818_347	4.52	9.67	1.96 (LK)	LK/FF	Kosgey et al. (2021)
DZ 2017	*QSr.cdl.4AL*	2.63	4A2	CAP12_c2972_140	wsnp_BG313770B_Ta_1_1	Kukri_c17417_407	4.30	16.62	2.47 (LK)	LK/LM	Edae et al., 2018
DZ 2017	*QSr.cdl.4DS*	53.00	4D	–	wsnp_Ex_c34252_42593715	RAC875_c67855_529	3.10	11.18	1.78 (LK)	LK/LM	New
DZ 2017	*QSr.cdl.5BL*	125.91	5B	CAP12_c2189_159	BS00022662_51	RFL_Contig4205_679	3.55	13.11	-1.98 (LM)	LK/LM	Crossa et al., 2007; Bansal et al., 2008; Bhavani et al., 2011
DZ 2018	*QSr.cdl.4DS*	52.0	4D	–	wsnp_Ex_c34252_42593715	RAC875_c67855_529	5.44	9.04	1.68(LK)	LK/LM	New
DZ 2018	*QSr.cdl.6A*	59.57	6A	IACX3586	Ra_c8185_676	Excalibur_c26057_1049	3.75	3.03	-1.50 (LM)	LK/LM	Babiker et al., 2017; Bajgain et al., 2015a
Ken 2017	*QSr.cdl.1AS*	25.0	1A	–	Kukri_c56494_585	BobWhite_c20553_364	3.29	11.47	-0.97 (FF)	LK/FF	Yu et al., 2011; Bajgain et al., 2015, Bhavani et al., 2011
	*QSr.cdl.2BS*	0.0	2B	Tdurum_contig29563_109	Tdurum_contig29563_109	GENE-0818-347	3.14	11.04	0.94 (LK)	LK/FF	Kosgey et al. (2021)
Ken 2018	*QSr.cdl.2BS*	1.86	2B	BS00064164_51	Tdurum_contig29563_109	GENE-0818-347	4.37	9.02	0.95 (LK)	LK/FF	Kosgey et al. (2021)
Ken 2018	*QSr.cdl.1BL*	11.1	1B2	BS00029539_51	Ra_c40444_243	BobWhite_c27474_154	3.02	4.46	2.67 (LK)	LK/LM	New
2018	*QSr.cdl.4DS*	32.0	4D	–	wsnp_BF473052D-Ta-_2_-1	Kukri_c159-61-70	4.12	1.57	1.63 (LK)	LK/LM	New
Ken 2018	*QSr.cdl.5BL*	128.01	5B	Kukri_c57954_369	Tdurum_contig58442_188	RFL_Contig4205_679	4.09	3.33	-2.20 (LM)	LK/LM	Crossa et al., 2007; Bansal et al., 2008; Bhavani et al., 2011

* Source parent of resistant allele is indicated in parenthesis.

**Table 5:** QTL identified for North American domestic stem rust races in Linkert/Forefront (LK/FF) and Linkert/LMPG-6 (LK/LM) populations evaluated at Rosemount, MN.

Population	Race	Year	QTL name	Chr	QTL pos (cM)	Marker @ QTL	Left Flanking	Right flanking	LOD	Permutation LOD (5%) threshold	Additive effect *	%Variance explained	Comments
LK/FF	QTHJC	2016	*QSr.cdl.1DS*	1D	7.0	–	IAAV5858	BS00066446_51	3.89	1.81	-1.07 (FF)	6.79	Edae et al., 2018; Bajgain et al., 2015a.
			*QSr.cdl.2BS*	2B	6.0	–	wsnp_Ku_c48_103915	GENE-0818_347	10.80	1.81	2.78 (LK)	7.59	Kosgey et al. (2021)
		2017	*QSr.cdl.3AS*	3A	9.19	wsnp_Ex_c12850_20377830	RAC875_c61343_250	–	1.86	1.81	0.78 (LK)	5.40	Letta et al., 2013
LK/FF	QFCSC	2015	*QSr.cdl.7BL*	7B	144.0	–	BS00085556_51	BS00077956_51	49.4	1.04	-0.05 (FF)	1.52	Crossa et al., 2007; Yu et al., 2011; Edae et al., 2018
		2016	*QSr.cdl.7BS*	7B	21	–	Ex_c3265_2068	IACX198	3.57	1.04	-0.63 (FF)	3.80	Haile et al., 2012
		2017	*QSr.cdl.3BS*	3B	27.3	wsnp_JD_c10233_10936535	wsnp_Ku_c17718_26860963	wsnp_JD_c10233_10936535	107.53	1.04	-1.54 (FF)	3.94	Yu et al., 2011; Bajgain et al., 2015a
LK/LM	QFCSC	2015	*QSr.cdl.1BL*	1B2	6.0	–	Ra_c40444-243	BobWhite_c2092-519	40.01	1.20	-213.81 (LM)	63.99	Bhavani et al., 2011
		2016	*QSr.cdl.1BL*	1B2	12.0	–	Ra_c40444_243	RFL_Contig5906_387	3.12	1.20	223.37 (LK)	68.99	Bhavani et al., 2011
		2016	*QSr.cdl.3BS*	3B	13.72	Excalibur_c45968_83	Excalibur_c45968_83	Excalibur_c45968_83	8.01	1.20	12.00 (LK)	3.14	Yu et al., 2011; Bajgain et al., 2015a
		2017	*QSr.cdl.4DS*	4D	36.00	–	wsnp_Ex_c34252_42593715	Kukri_c15961_70	41.23	1.20	7.92 (LK)	38.36	New
LK/FF	RCRSC	2017	*QSr.cdl.1BS*	1B	52.0	–	RAC875_c25125_210	GENE-0165_389	14.12	2.85	0.034 (FF)	4.85	Crossa et al., 2007
			*QSr.cdl.3AL*	3A	96.0	–	RAC875_rep_c109228_400	IAAV9044	23.6	2.85	0.03 (LK)	2.93	Letta et al., 2013
LK/LM	RCRSC	2015	*QSr.cdl.5BL*	5B	122.23	BS00000848_51	Tdurum_contig47816_258	BS00080474_51	4.92	0.51	6.32 (LK)	1.03	Bajgain et al., 2015a; Edae et al., 2018
		2015	*QSr.cdl.1BL*	1B2	10.57	BobWhite_c2092_519	Ra_c40444_243	BobWhite_c27474_154	2.48	0.51	-6.07 (LM)	1.81	Edae et al., 2018
		2016	*QSr.cdl.3AL*	3A2	5.82	BobWhite_c11935_137	RAC875_c15390_459	BS00004149_51	5.82	0.51	7.50 (LK)	1.28	Edae et al., 2018
		2016	*QSr.cdl.4AL*	4A2	2.63	CAP12_c2972_140	wsnp_BG313770B_Ta_1_1	Kukri_c17417_407	2.63	0.51	18.21 (LK)	1.97	Edae et al., 2018; Kosgey et al., 2021
		2017	*QSr.cdl.5BL*	5B	121.69	Excalibur_rep_c105964_928	Ex_c67086_584	Excalibur_rep_c88310_1394	4.12	0.51	11.23 (LK)	1.94	Bajgain et al., 2015a; Megerssa et al., 2020
LK/FF	TPMKC	2015	*QSr.cdl.7AL*	7A	157.0	–	wsnp_Ex_c8692_14557179	Ra_c9427_300	18.57	1.71	7.16 (LK)	10.61	Yu et al., 2011; Bajgain et al., 2015a; Edae et al., 2018
											
		2016	*QSr.cdl.2AS*	2A	8.0	–	GENE-1177_195	–	2.75	1.71	8.45 (LK)	17.32	Juliana et al., 2019
		2017	*QSr.cdl.1BL*	1B	79.00	Tdurum_contig81102_102	Tdurum_contig57731_412	Tdurum_contig81102_102	3.08	1,71	-0.17 (FF)	2.97	Bhavani et al., 2011; Edae et al., 2018
LK/LM	TPMKC	2015	*QSr.cdl.4BS*	4B	45.00	–	RAC875_c2542_815	BobWhite_c42663_70	63.8	0.74	0.60 (LK)	2.76	Crossa et al., 2007; Bajgain et al., 2015b; Chao et al., 2017
		2016	*QSr.cdl.1BS*	1B	0.0	wsnp_RFL_Contig3951_4390396	BobWhite_c23617_167	TA003668-0364	2.74	0.74	-8.32 (LM)	5.04	Crossa et al., 2007; Yu et al., 2011, Shewabez et al., 2022; Bajgain et al., 2015a
		2016	*QSr.cdl.1BL*	1B2	6.00	–	BS00022323_51	BobWhite_c2092_519	2.76	0.74	-2.71 (LM)	1.97	Chao et al., 2017; Edae et al., 2018
		2016	*QSr.cdl.5AS*	5A	47.0	Tdurum_contig82190_124	–	–	2.39	0.74	-8.67 (LM)	6.31	Chao et al., 2017
		2016	*QSr.cdl.5BL*	5B	123.8	BS00080474_51	wsnp_Ex_c58091_59534826	RFL_Contig4205_679	2.01	0.74	7.32 (LK)	6.18	Bajgain et al., 2015a; Megerssa et al., 2020
		2016	*QSr.cdl.7AS*	7A	27.02	Ku_c6386_1034	CAP12_c2951_105	Excalibur_c8066_791	2.79	0.74	7.60 (LK)	4.56	Bajgain et al., 2015b; Chao et al., 2017; Edae et al., 2018

*Source parents of resistant allele are indicated in parathesis.

**Table 6:** Seedling QTL for domestic races and race TKTTF based on infection type (IT) for Linkert/Forefront (LK/FF) and Linkert/LMPG-6 (LK/LM) populations.

Race	QTL name	QTL position	Chr	Marker @ QTL	Left Flanking	Right flanking	LOD	Permutation LOD (5%) threshold	%Variance explained	Additive effect *	Population	Comments
QTHJC	*QSr.cdl.7BS*	43.0	7B	–	IACX198	wsnp_Ex_c2103_3947695	3.06	3.03	8.96	0.95 (LK)	LK/FF	New
TPMKC	*QSr.cdl.2BS*	1.86	2B	BS00064164_51	Tdurum_contig29563_109	GENE-0818_347	4.21	3.03	2.30	0.01 (LK)	LK/FF	Kosgey et al. (2021)
TPMKC	*QSr.cdl.7BS*	15.04	7B	BobWhite_c47269_128	Ex_c3265_2068	IACX198	4.73	3.03	3.43	-0.07 (FF)	LK/FF	Kosgey et al. (2021)
RCRSC	*QSr.cdl.1BS*	51.0	1B	–	RAC875_c25125_210	BS00062740_51	4.63	3.03	1.28	0.18 (FF)	LK/FF	Crossa et al., 2007
QFCSC	*QSr.cdl.3BS*	19.00	3B	BS00089954_51	Excalibur_c45968_83	wsnp_Ku_c1391_2771050	7.97	2.87	2.18	0.19 (LK)	LK/LM	Yu et al., 2011; Bajgain et al., 2015a
RCRS	*QSr.cdl.2AS*	6.85	2A	Ra_c58279_702	BS00073381_51	Excalibur_c51876_189	4.16	2.87	3.60	-0.25 (LM)	LK/LM	Juliana et al., 2019
RCRS	*QSr.cdl.4AL*	2.63	4A2	CAP12_c2972_140	wsnp_BG313770B_Ta_1_1	Kukri_c17417_407	7.63	2.87	3.01	-0.20 (LM)	LK/LM	Edae et al., 2018; Kosgey et al., 2021
TKTTF	*QSr.cdl.4AL*	2.11	4A2	Excalibur_c19666_778	wsnp_BG313770B_Ta_1_1	Kukri_c17417_407	9.31	2.99	24.902	1.60 (LK)	LK/LM	Edae et al., 2018; Kosgey et al., 2021

*Source parents of resistant allele are indicated in parenthesis.

**Table 7:** QTL identified for Linkert/MN07098-6 population evaluated at Kenya and Ethiopia for two seasons.

Environment	Chr	QTL pos (mbp)	Marker @QTL	Left flanking	Right flanking	LOD	Additive effect	PEV (%)	Comments
ETH2016 (MS)	2B	30.1	chr2B_30100675	chr2B_28328844	chr2B_40354375	8.27	6.10	19.32	Kosgey et al. (2021)
	4B	506.3	–	*	*	3.24	-0.99	1.54	Crossa et al., 2007; Bajgain et al., 2015b; Chao et al., 2017
Ken2016 (MS)	2B	32.9	–	chr2B_30100675	chr2B_40354375	16.11	5.40	16.15	Kosgey et al. (2021)
	3A	568.3	–	*	*	4.40	0.44	0.54	Letta et al., 2013; Edae et al., 2018
	3D	109.9	–	*	*	6.50	1.16	0.62	New
	4A	552.4	–	*	*	4.98	-0.38	0.70	Edae et al., 2018; Kosgey et al., 2021
	4B	527.3	–	*	*	4.86	0.35	0.01	Bajgain et al., 2015b
Ken2017 (OS)	2A	638.6	–	*	*	3.64	0.75	0.63	Bajgain et al., 2015a; Edae et al., 2018
	2B	28.9	–	chr2B_24907176	chr2B_40354375	14.40	5.78	7.40	Edae et al., 2018
	3B	104.3	–	*	*	3.71	0.63	0.53	New
	3D	328.9	–	*	*	4.15	-0.39	0.02	Edae et al., 2018
	4A	520.4	–	*	*	4.73	-0.47	0.13	Edae et al., 2018
	4B	78.3	–	*	*	4.08	-0.13	0.11	New
	4D	287.9	–	*	*	3.89	-0.08	0.15	McIntosh et al., 2012
	5A	401.7	–	*	*	4.62	-0.02	0.40	Bajgain et al., 2015a; Chao et al., 2017; Edae et al., 2018
	6D	133.2	–	*	*	3.81	0.39	0.70	Bajgain et al., 2015b
	7B	345.4	–	*	*	3.89	0.42	0.03	New

-= No marker at the identified QTL; * Flanking marker are far away from the identified QTL

A majority of the QTL were population, environment, and race-specific. However, one QTL (marker: CAP12_c2189_159) on chromosome 2BS (1.9 cM) was detected across two populations in multiple environments (**Tables 4**, **7**). It was also detected for field response to race QTHJC and seedling response to race TPMKC (**Tables 5**, **6**). A population-specific stable QTL was detected on chromosome 5BL (125.9 cM) in the LK/LM population across environments in Ethiopia and Kenya (**Table 4**). It also provided field resistance against races RCRSC and TPMKC (**Table 5**). The gene *Sr7a* was detected on 4AL (marker: CAP12QT_c2972_140) in the LK/LM population in Ethiopia and in Rosemont for response to RCRSC in 2016, and seedling response to race TKTTF. In Linkert/MN07098-6, a QTL on chromosome 2BS (30.1 Mbp) was consistently detected and explained up to 19.3% of the phenotypic variation (**Table 7**). At chromosome level, Linkert/MN07098-6 had a total 11 chromosomes on which QTL were detected and some of the QTL had similar chromosome regions (e.g., 2BS) as detected in other populations.

## Discussion

The objective of this study was to characterize the genetics of stem rust resistance in the hard red spring wheat variety Linkert by testing progenies developed by crossing with three stem rust susceptible lines, cultivar Forefront, breeding line MN07098-6, and genetic stock LMPG-6, with different *Pgt* races in the field and at the seedling stage in a greenhouse.

A reliable stem rust QTL was identified on chromosome 2BS in the Linkert/Forefront and Linkert/MN07098-6 populations. Interestingly, in addition to conferring adult plant resistance to the Ug99 race group in African field environments, it was detected at the seedling stage for response to race TPMKC. In addition to relatively consistent expression under different environments, the phenotypic variation explained by the QTL was also substantial (9.02-19.32%). Seven *Sr* genes have been reported on 2BS, and out of these *Sr36, Sr39*, and *Sr40* (Wu et al., 2009; Niu et al., 2011; Rouse et al., 2012) are effective against the Ug99 race TTKSK. However, the QTL detected in this study is only effective to the Ug99 race group at the adult growth stage, excluding the previously described major effect all-stage resistance genes on chromosome arm 2BS. The coincidence of this QTL with seedling response to race TPMKC warrants further study. Kosgey et al. (2021) also detected a QTL (*QSr.cdl-2BS.2*) on 2BS using field-tested recombinant inbred lines derived from a cross made between CI 14275 (resistant parent) and LMPG-6 (susceptible parent), and the KASP marker that was developed from SNP Excalibur_c7963_1722_C1 was also associated with reduced stem rust severity. Thus, the resistance in Linkert and CI 14275 is most likely similar. Previous QTL mapping studies have also shown that there were at least eight QTL regions on the short arm of chromosome 2B (from 0-160 mbp range) detected in both GWAS and bi-parental populations (Crossa et al., 2007; Bajgain et al., 2015a; Bajgain et al., 2015b; Bajgain et al., 2016; Edae et al., 2018), and the associated SNPs were IWB2369 (pos: 48.5 cM; 31.50 mbp), IWB69830 (pos: 46.76 cM; 31.30 mbp), IWB24614 (104. 80 mbp), IWB32327 (104.80 mbp), IWB23439 (48.04 cM; 55.04 mbp), and BS00073426-51 (160.00 mbp). Gene *Sr23* mapped on distal end of 2BS and was described as completely linked with *Lr16* (McIntosh and Luig, 1973b) and expressed under conditions of high temperature and high light intensity (Luig, 1983). All cultivars that possess *Lr16* also were reported to possess *Sr23* (Kassa et al., 2017). Cultivars such as Exchange, Selkirk, Warden and Etoile de choisy are sources of *Sr23*. A recent report also indicated that *Lr16* is present in North American cultivars such as ‘AC Domain’, ‘AC Karma’, ‘AC Majestic’, ‘AC Splendor’, ‘Columbus’, and ‘Grandin’ (Kassa et al., 2017). Although major gene *Lr16/Sr23* was mapped on chromosome 2BS, it is unlikely that the QTL detected in the current study is *Lr16/Sr23* because the QTL is mapped 21 cM away from that of the *Lr16/Lr23* position.

The Linkert/LMPG-6 population possessed a relatively consistent QTL on chromosome 4AL (SNP: CAP12_c2972_140) that provided resistance against different *Pgt* races both at seedling and adult stages implying it can provide all-stage resistance against stem rust races RCRSC and TKTTF. Edae et al., 2018 reported a strong association signal in the region of major gene *Sr7a* for resistance against race RCRSC. Similarly, in this study the QTL on chromosome 4AL was detected at adult and seedling stages for *Pgt* race RCRSC, indicating Linkert possesses gene *Sr7a*, as previously postulated (Anderson et al., 2018).

The QTL detected on chromosome 5BL in Linkert/LMPG-6 was relatively stable. This region of 122-128 cM on the long arm of chromosome 5B harbored a QTL that provided field resistance against virulent races in East Africa (Ethiopia and Kenya) and North American *Pgt* races (RCRSC and TPMKC). This QTL was detected in the Linkert/LMPG-6 population but not in the Linkert/Forefront or Linkert/MN07098-6 populations. Stem rust resistance gene *Sr56* that confers adult plant resistance (APR) was mapped on chromosome 5BL (McIntosh et al., 2012). Several QTL have been reported using both bi-parental populations and GWAS including adult plant resistance gene *Sr56* (Crossa et al., 2007; Bansal et al., 2008; Bhavani et al., 2011). In previous studies the 5BL QTL representing *Sr56* explained 10-13% phenotypic variation and contributed an important component of the “*Sr2* complex” (Bhavani et al., 2011). The QTL detected in the current study explained up to 13% of phenotypic variation, and the QTL, *QSr.Sun-5BL*, reported by Bansal et al., 2008 also explained 11 to 12% of the phenotypic variation in adult plant stem rust response and was responsible for a 12-15% reduction in stem rust severity. Since *Sr56* was first reported from European ‘Arina’ winter wheat, a pedigree link between *Sr56* and Linkert is not known. Further studies are warranted to determine if the 5BL QTL is *Sr56*. It is possible that both Forefront and MN07098-6 also possess the resistance allele of the 5BL QTL, which would explain why it was not detected in the corresponding populations.

Besides stem rust QTL on 2BS, 5BL, and 4AL, numerous environment and population-specific QTL were found that corresponded with previously reported QTL. Three QTL were detected on chromosome 7A. Two of them provided resistance against *Pgt* race TPMKC (pos: 157.0 cM and 27.0 cM). Several QTL have been reported on 7AL (Bajgain et al., 2015a; Edae et al., 2018) for stem rust resistance, and the major genes *Sr15* and *Sr22* are located on 7AL (Sears and Briggle, 1969; The and McIntosh, 1975). Similarly, QTL were detected on chromosome 7B for stem rust resistance. Two QTL that provided field resistance against races QTHJC (pos: 126.0 cM) and QFCSC (pos: 144.0 cM) were detected on 7BL. Edae et al., 2018 also reported QTL for *Pgt* race QFCSC on 7BL (pos: 109.0 cM) using association mapping.

The seedling and single-race field experiments in Rosemount, MN facilitated a precise evaluation of resistance QTL to the four races evaluated. As expected, all-stage resistance gene *Sr7a* was consistently detected in both seedling and field studies in response to avirulent race RCRSC. However, the other QTL were detected only in seedling or field environments. This seems to contrast with the expectation that QTL detected at the seedling stage would be effective in the field consistent with “all-stage” resistance. This may be explained by the relatively low effectiveness of the seedling QTL detected other than *Sr7a*. Not detecting these weakly effective QTL in the field may be the result of (1) true ineffectiveness of these QTL in the field or (2) the masking of weakly effective all-stage QTL by the presence of adult plant resistance loci. Our results are similar with the findings of an association mapping study conducted by Edae et al., 2018 where conventional North American spring wheat lines possesses largely seedling- or field-effective QTL. Only the most strongly effective QTL were detected in both seedling and field studies.

In conclusion, despite the large number of environment and race-specific QTL identified in the current study, we found Linkert derived QTL that were consistently associated with adult plant resistance to Ug99 stem rust on chromosome 2BS in the Linkert/Forefront and Linkert/MN07098-6 populations. Additional QTL were detected consistently on chromosomes 5BL (125.9 cM) and 4AL (*Sr7a*) with the Linkert/LMPG population. Combined with the detection of the QTL on 2BS in previous studies, the identification of the 2BS QTL in the two populations derived from conventional germplasm demonstrates the importance of this QTL in contributing towards stem rust adult plant resistance in United States hard red spring wheat.

## Data availability statement

The original contributions presented in the study are included in the article/**Supplementary Materials**, further inquiries can be directed to the corresponding authors.

## Author contributions

EE: Conceptualization, Data curation, Formal analysis, Investigation, Methodology, Project administration, Software, Validation, Visualization, Writing – original draft, Writing – review & editing. ZK: Writing – review & editing. PB: Data curation, Writing – review & editing. KN: Conceptualization, Data curation, Investigation, Writing – review & editing. AG: Data curation, Methodology, Writing – review & editing. SB: Resources, Writing – review & editing. JA: Funding acquisition, Project administration, Resources, Writing – review & editing. MR: Conceptualization, Funding acquisition, Investigation, Project administration, Resources, Supervision, Writing – review & editing.
