# Supplementary material for: The genetics of Ug99 stem rust resistance in spring wheat variety ‘Linkert‘
Source: Front Plant Sci. 2024 Mar 7;15:1343148. doi: 10.3389/fpls.2024.1343148 (PMC10954791; doi:10.3389/fpls.2024.1343148)
Supplement: Supplementary file 3 [file DataSheet_3.pdf]

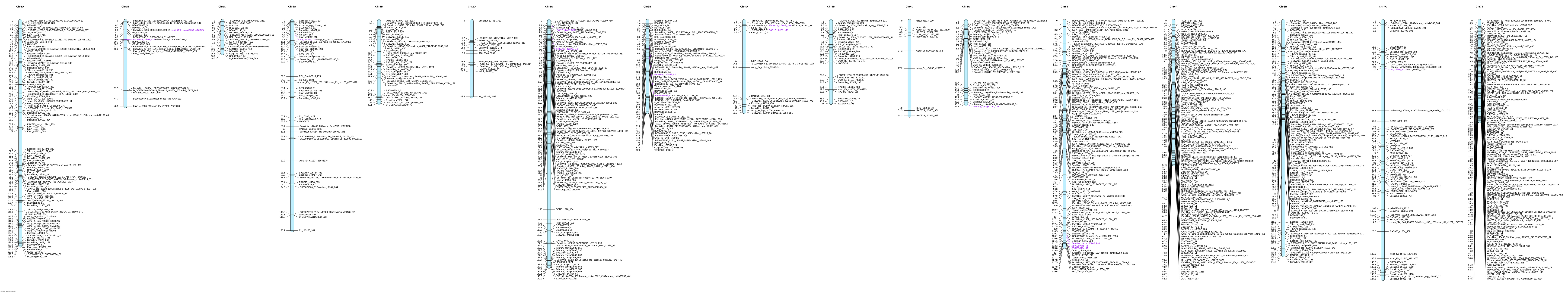
Chr1A
Chr1B
Chr1D
Chr2A
Chr2B
Chr2D
Chr3A
Chr3B
Chr4A
Chr4B
Chr4D
Chr5A
Chr5B
Chr6A
Chr6B
Chr7A
Chr7B
